# Tip60 HAT Action Mediates Environmental Enrichment Induced Cognitive Restoration

**DOI:** 10.1371/journal.pone.0159623

**Published:** 2016-07-25

**Authors:** Songjun Xu, Priyalakshmi Panikker, Sahira Iqbal, Felice Elefant

**Affiliations:** Department of Biology, Drexel University, Philadelphia, PA, United States of America; Oregon Health and Science University, UNITED STATES

## Abstract

Environmental enrichment (EE) conditions have beneficial effects for reinstating cognitive ability in neuropathological disorders like Alzheimer’s disease (AD). While EE benefits involve epigenetic gene control mechanisms that comprise histone acetylation, the histone acetyltransferases (HATs) involved remain largely unknown. Here, we examine a role for Tip60 HAT action in mediating activity- dependent beneficial neuroadaptations to EE using the *Drosophila* CNS mushroom body (MB) as a well-characterized cognition model. We show that flies raised under EE conditions display enhanced MB axonal outgrowth, synaptic marker protein production, histone acetylation induction and transcriptional activation of cognition linked genes when compared to their genotypically identical siblings raised under isolated conditions. Further, these beneficial changes are impaired in both Tip60 HAT mutant flies and APP neurodegenerative flies. While EE conditions provide some beneficial neuroadaptive changes in the APP neurodegenerative fly MB, such positive changes are significantly enhanced by increasing MB Tip60 HAT levels. Our results implicate Tip60 as a critical mediator of EE-induced benefits, and provide broad insights into synergistic behavioral and epigenetic based therapeutic approaches for treatment of cognitive disorder.

## Introduction

Alzheimer’s disease (AD) is the most common form of dementia in the aging population and its progression is tightly associated with cognitive impairments that involve learning and memory deficits. The pathology of AD has been linked to neuronal cell death and disrupted synaptic plasticity in various brain regions that specifically include the hippocampus and the cortex. Increasing compelling evidence demonstrates that AD progression is influenced by a complex interplay between genetic and environmental risk factors, and that such gene-environmental interactions play a major role in triggering pathways that can either slow or exacerbate disease progression. Environmental stimuli provide neurons in the brain with instructive information that shapes synaptic connections to impact cognitive ability. As such, environmental enrichment (EE) conditions have beneficial effects for reinstating cognitive ability in neuropathological conditions such as AD. EE has been shown to enhance hippocampal neurogenesis and reverse learning and memory deficits by inducing structural changes in the neuronal network to enhance synaptic efficacy. While a substantial body of evidence demonstrates that EE benefits involve epigenetic gene control mechanisms that comprise histone acetylation induction, the select HATs involved and their mechanisms of action underlying this process remain largely unknown.

We previously demonstrated that Tip60 HAT action controls activity-dependent cognition-linked neuronal processes that include synaptic plasticity, axonal transport and outgrowth, learning and memory and epigenetically regulates transcriptional profiles of genes enriched for these functions. Consistent with a role for Tip60 in nervous system function, our laboratory [1–10] and others [2, 3, 11–13] have demonstrated that Tip60 is implicated in Alzheimer’s disease (AD) based on its role in epigenetic neuronal gene control *via* its formation of a transcriptionally active complex with the processed C- terminal amyloid precursor protein (APP) intracellular domain (AICD) [2, 11, 12, 14] [7, 15–19]. We further made the exciting discovery that increasing *in vivo* Tip60 HAT levels in the *Drosophila* nervous system under APP induced neurodegenerative conditions rescues AD associated neuronal impairments such as apoptotic neurodegeneration in the central nervous system (CNS) [7], axonal outgrowth [5, 6] and synaptic vesicle transport in motor neurons[2]. Excess Tip60 also restores associated disrupted complex functional abilities impaired in AD that include sleep cycles[5, 6], locomotor function[2] and learning and memory[10] defects with concomitant induction of some genes critical for the function of these neural processes [2, 7]. In direct contrast, loss of Tip60 HAT function in the fly nervous system causes gene misregulation and exacerbates such AD associated impaired phenotypes [2, 5–7, 10] Together, our findings demonstrate that Tip60 plays a neuroprotective role in an array of cognition associated neuronal processes that are impaired during the early stages of the AD pathological process.

Environmental enrichment (EE) conditions comprising positive social reinforcements has also been shown to have neuroprotective benefits under neuropathological conditions such as AD [20–23]. While experimental EE conditions may vary between studies exploring EE neuroadaptative benefits, one critical and non-variable EE component widely conserved amongst species is social environmental enrichment [24, 25]. Well established studies using *Drosophila* show that similar to mammals, social EE promotes significant beneficial structural changes in regions throughout the fly brain that include the mushroom body (MB) that regulates a variety of behavioral and physiological functions ranging from olfactory learning and memory to decision making under uncertain conditions[26–30]. Social EE promotes enhanced MB axon and dendrite formation, synaptic plasticity and neuronal MB Kenyon cell growth[24, 31]. Recent studies demonstrate that EE benefits require epigenetic gene regulation involving induction of specific histone acetylation profiles [21, 32–34]. Nevertheless, how specific HATs mediate cognitive gene expression programs in response to changing environmental cues and the select HATs involved in this process remain largely unknown.

Here, we exploit the power of *Drosophila* genetics and the behavioral and physiological conservation between flies and mammals in terms of their positive neuroadaptive response to ask whether Tip60 HAT action is required for an EE induced beneficial neuroadaptative response. We use the MB as our well characterized cognitive model as this neural circuit in the adult fly brain is where Tip60 is robustly produced. As the central for learning and memory, MB exhibits beneficial morphological changes in response to EE similar to mammalian systems[25, 35–37]. Our findings implicate Tip60 as a critical mediator of EE-induced benefits, and provide broad insights into non-invasive synergistic behavioral and epigenetic approaches for treatment of cognitive deficits in neurological disorders.

## Results

### Tip60 HAT action restores EE mediated neuroadaptative benefits under APP neurodegenerative conditions

**Fig 1:**
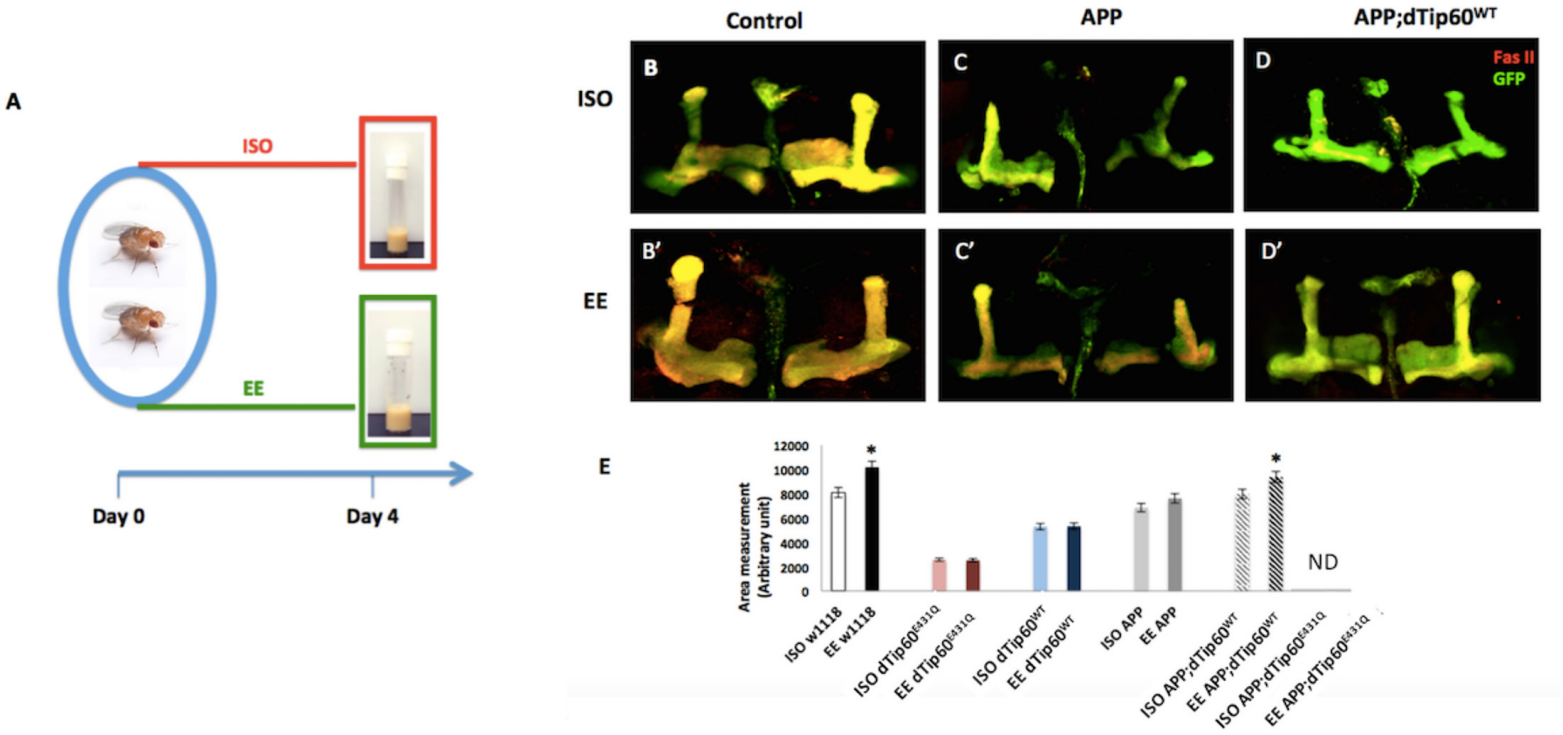
Tip60 HAT activity restores EE mediated neuroadaptive benefits on MB morphology under APP neurodegenerative conditions. (A) Experimental paradigm for flies exposed to isolation (ISO) or environmental enrichment (EE) conditions. Newly eclosed adult fly progeny were exposed to a group of 30 flies (1:1 sex ratio) (EE), or housed individually (ISO) for 5 days. (B-D) Representative confocal images of adult MB visualized by mCD8-GFP and stained with axonal marker Fascillin II (Fas II) antibody from 5-day old adult fly expressing indicated transgenes driven by GFP;;OK107-Gal4 under ISO or EE condition. Anti-GFP staining used as marker to delineate MB in the adult brains. Anti-Fas II staining shows a/ß and r lobes in wild-type, APP and APP;dTip60^WT^ flies under ISO condition. (B’-D’) Same genotype flies in EE condition. (E) Quantification of area in the different genotypes and housing conditions in adult flies. Data represent the mean of 15 replicates with error bars depicting 95% confidence interval. Student t-test was used to determine statistical significance between different housing conditions within the same genotype. APP;dTip60^E431Q^ flies exhibited severely malformed MB structure and lack of structural consistency, and therefore are labeled as ND (non-determined) in the quantification. ** P < 0.01, *P < 0.05.

We previously demonstrated that Tip60 is required for both MB morphology and function in learning and memory[10, 38]. Thus, we first asked whether EE promotes beneficial changes on MB structural morphology and whether this response is dependent upon Tip60 HAT action. To test this, we crossed flies carrying a UAS-mCD8-GFP marker in conjunction with the MB specific OK107-GAL4 driver to control w^1118^ flies to mark MB cells with GFP for enhanced visualization. The MB GAL4 driver OK107 expressed GAL4 in discrete neuronal populations in the adult fly brain that includes high expression in the Kenyon cells, the intrinsic neurons of the MB as well as in the pars intercerebralis, suboesophageal ganglion and optic lobes [26]. To assess MB response to EE, newly eclosed adult fly progeny were exposed to environmental enrichment conditions (EE) or isolation conditions (ISO) following established protocols[24, 39, 40]. Briefly, newly eclosed flies were either exposed to a group of 30 flies (1:1 sex ratio) (EE) or housed individually (ISO) for 5 days (Fig 1A). MBs from dissected conditioned brains were stained with antibodies to GFP to delineate the MB, counterstained with axonal marker FasII antibody that exhibits weak expression in the γ lobe while strongly labeling a/ß lobes, and MB area was quantitated for each genotype (Fig 1B–1D, 1B’–1D’ and 1E). Quantification analysis on MB area reveals the EE conditioned MB structure is significantly larger than MB from ISO siblings that were housed individually (Fig 1B and 1B’; P<0.01), consistent with previous studies [23, 32, 41] [36, 37]showing that the MB undergoes an EE induced beneficial neuroadaptive response. To assess the role of Tip60 HAT activity in this MB EE neuroadaptative response, we first asked whether modulating Tip60 HAT levels in the MB would alter the EE response we observe in control w^1118^ flies. To test this, we used our laboratory’s previously generated GAL4-responsive transgenic fly lines carrying a membrane-bound mCD8-GFP construct with either Tip60 dominant negative HAT mutant (UAS-mCD8-GFP;dTip60^E431Q^) or wild-type Tip60 (UAS-mCD8-GFP;dTip60^WT^)[5] and crossed them to MB driver OK107. Newly eclosed adult fly progeny for each genotype were exposed to EE or ISO conditions (Fig 1A) [24, 39, 40] and MBs from dissected conditioned brains were stained with antibodies to GFP to delineate the MB, counterstained with axonal marker FasII, and MB area was quantitated for each genotype (Fig 1E). Quantitation analysis demonstrated that, consistent with our previous findings, dTip60^E431Q^ flies showed a significant reduction in MB total area when compared to control w^1118^ flies while dTip60^WT^ flies display a less severe reduction in MB total area (S1 Fig). Additionally, both loss and gain of Tip60 HAT levels in the fly MB resulted in a lack of response to EE (Fig 1E and S1 Fig). Our results suggest that appropriate levels of Tip60 HAT activity are required for EE mediated neuroadaptative morphological benefits.

EE has been shown to enhance cognitive ability under neuropathological conditions such as Alzheimer’s disease (AD) [20–23] *via* induction of histone acetylation, yet the full array of HATs involved remain to be identified[12, 42, 43]. Thus, we wished to ask whether EE promotes beneficial changes on MB morphology under AD associated APP neurodegenerative conditions and whether this response is dependent upon Tip60 HAT action. To examine the effects of EE on MB morphological changes under APP induced neurodegenerative conditions, we used unique UAS-responsive transgenic fly lines generated in our laboratory[7] that co-express wild type Tip60 (dTip60^WT^) and human APP driven by GFP;OK107-Gal4. This system allows us to manipulate Tip60 HAT levels in an APP neurodegenerative background, while simultaneously marking and visualizing the MB neurons using GFP. Flies from each cross were housed in ISO or EE conditions and the conditioned brains of 5-day-old adult animals were dissected, stained with antibodies to GFP to delineate the MB, counterstained with axonal marker FasII, and the MB area was quantitated for each genotype (Fig 1B–1E). This analysis revealed that while the overall stereotypical morphology of MB lobes was detected in APP expressing fly lines, both a/ß and a’/ß’ lobes were significantly thinner and shorter in both EE and ISO conditioned APP MB when compared to control flies (OK107-GAL4/UAS-GFP) (Fig 1C–1C’). Quantitative analysis of the MB lobe area revealed a non-significant increase in MB area in APP flies under EE conditions, indicating that the EE induced beneficial neuroadaptative response we observe in control flies (Fig 1B–1B’ and 1E) is compromised under APP neurodegenerative conditions.

Given our previous findings that increased levels of Tip60 rescues multiple neural circuits impaired under APP induced neurodegenerative conditions while Tip60 HAT loss exacerbates APP defects [5, 7, 10], we asked whether increased Tip60 HAT levels could also restore the impaired EE response we observe in APP MB. To test this, we crossed flies carrying a UAS-mCD8-GFP marker in conjunction with MB specific OK107-GAL4 driver to either control w^1118^, APP;Tip60^WT^ or APP;Tip60^E431Q^ flies to simultaneously tag MB cells with GFP while increasing or decreasing Tip60 HAT levels in the MB under APP neurodegenerative conditions. We found that adult brains from APP;dTip60^WT^ fly showed no observable MB structural defects as assessed by GFP and Fas II labeling of a/ß and a’/ß’ and r lobes, indicating increasing Tip60 HAT levels restored APP induced MB axonal growth defects (Fig 1D–1D’). Moreover, quantification analysis using FasII staining revealed a marked increase in the area of all 3 MB lobes in the APP;dTip60^WT^ flies in EE versus ISO conditions. In direct contrast, adult APP;dTip60^E431Q^ flies exhibited severe axonal defects in all three lobes and did not respond to EE conditions (Fig 1 and S1 Fig). Taken together, our result suggest that appropriate levels of Tip60 HAT activity are required for EE mediated neuroadaptative morphological benefits, and that excess Tip60 alleviates impairment of an EE response in APP flies.

### Tip60 restores EE induced positive changes in synaptic marker protein production

Our finding that increasing Tip60 HAT levels in the MB restores EE neuroadaptative benefits under APP neurodegenerative conditions prompted us to ask whether the beneficial MB structural changes we observed were accompanied by positive synaptic changes. To test this, we crossed flies carrying a UAS-mCD8-GFP marker in conjunction with MB specific OK107-GAL4 driver to either control w^1118^, APP or APP;Tip60^WT^ flies and assessed pre- and post-synaptic protein production using whole-brain homogenates from EE or ISO conditioned adult fly brains for each genotype.

**Fig 2:**
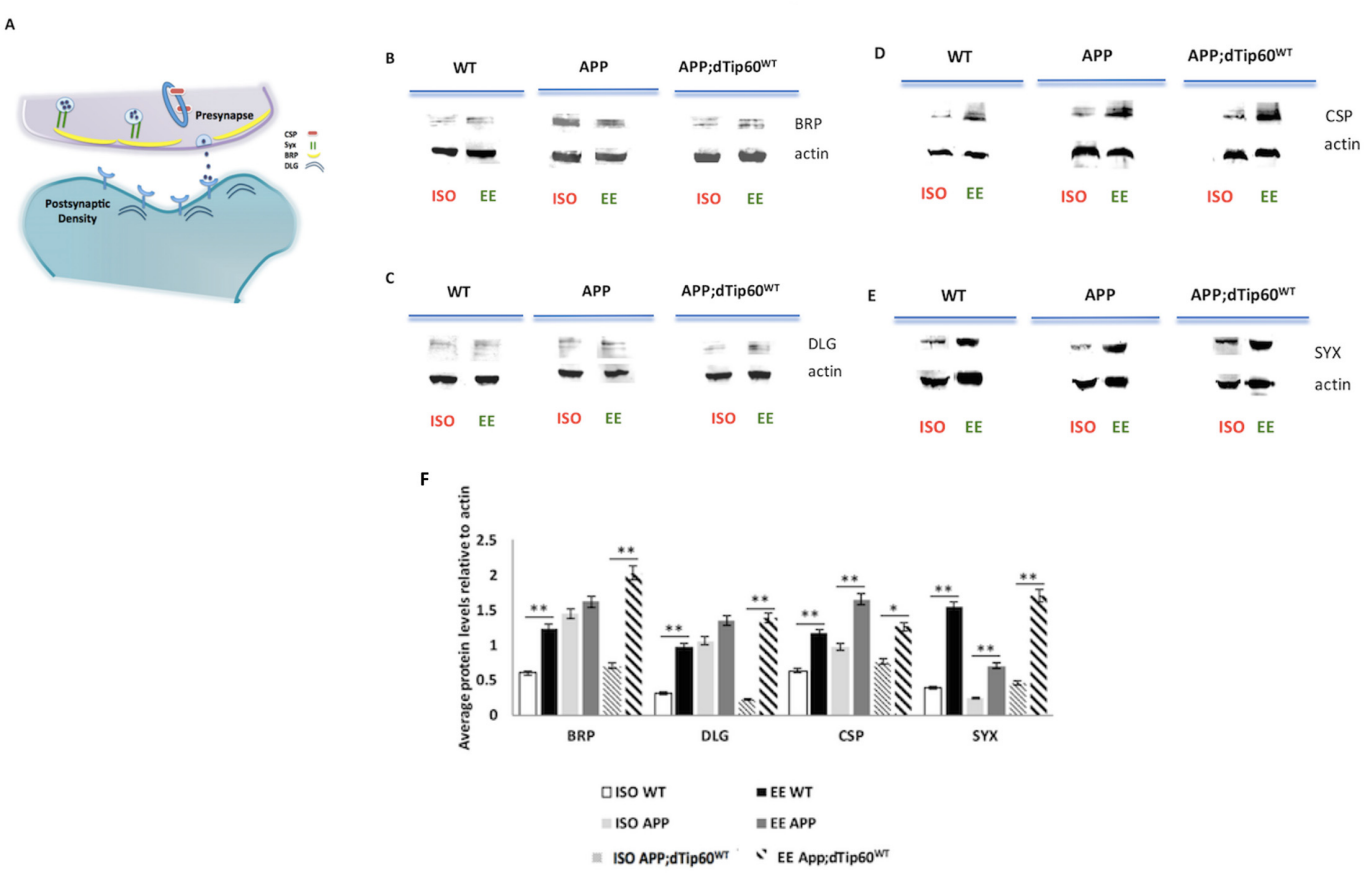
Tip60 restore EE induction of synaptic markers under APP neurodegenerative conditions. (A) The schematic of key synaptic maker components of *Drosophila* synapse. BRP, Bruchpilot; CSP, cysteine string protein; DLG, Discs-large; Syx, syntaxin. (B) Structural synaptic marker BRP and DLG protein production remained the same under different housing conditions under APP neurodegenerative conditions. (C) Structural marker protein production were induced under EE condition with enhanced Tip60 HAT activity. (D-E) Secretary marker protein Syx and CSP production were induced under EE conditions in both APP and APP;dTip60^WT^ fly brains. Data represent the mean of 3 replicates with error bars depicting 95% confidence interval. Student t-test was used to determine statistical significance between different housing conditions within the same genotype. ** P < 0.01, *P < 0.05.

To investigate pre-synaptic protein changes, we focused on Bruchpilot (BRP) (Fig 2A). BRP is a critical component in regulating the clustering of voltage gated calcium channels (VGCC) at the pre-synaptic active zone of all synapses, and as such, is commonly used as a marker for both synapse number and functionality in synaptic transmission [44–46]. Wild type w^1118^, APP and APP;dTip60^WT^ flies were subjected to EE and ISO conditioning. At the end of the 5 days, conditioned brains were dissected and BRP protein levels were assessed using quantitative analysis of Western blots. Consistent with our MB structural findings (Fig 1C–1E), APP;dTip60^WT^ flies exhibited enhanced BRP levels under EE conditions relative to ISO condition, while APP flies showed a non-significant change in BRP levels in response to EE (Fig 2B and 2F).

To assess post-synaptic protein changes, we focused on density marker Disc-large (DLG) that is primarily produced post-synaptically (Fig 2A). DLG is the *Drosophila* homolog of the post-synaptic mammalian density protein PSD-95/SAP-90 and is robustly produced in the CNS neuropil, where its protein localization pattern overlaps with that of BRP[47] [48]. DLG is involved in neurotransmitter release by regulating the post-synaptic clustering of glutamate receptors and controls glutamate release and post-synaptic structure [49–51]. Consistent with our MB structural and BRP findings (Figs 1C–1C’, 2B and 2F), APP;dTip60^WT^ flies exhibited enhanced DLG levels under EE conditions relative to ISO condition, while APP flies showed a non-significant change in DLG levels in response to EE (Fig 2C and 2F).

To assess levels of additional secretory machinery components, we analyzed the presence and distribution of two pre-synaptic vesicle associated proteins essential for synaptic transmission function: the cysteine string protein (csp) that regulates the activity of presynaptic Ca^2+^ channels to control exocytosis[52], and syntaxin (syx) a neuronal representative of a large family of proteins that promotes synaptic vesicle fusion and endocytosis mediated vesicle recycling, thus functionally “marking” both sides of the vesicle cycle (Fig 2A)[53, 54]. After EE conditioning, both CSP and SYX protein levels were found to increase (Fig 2D, 2E and 2F) in both APP and APP;dTip60^WT^ flies similar to that of wild-type w^1118^ flies, suggesting that unlike DLG and BRP, EE promotes secretary protein production in a Tip60 independent manner under APP induced neurodegenerative conditions. Taken together, these data suggest that Tip60 mediated enhancement of EE induced neuroadapative benefits under APP neurodegenerative conditions is due at least in part, to an increase in certain pre- and post-synaptic functional protein components.

### Tip60 promotes EE beneficial neuroadaptative transcriptional changes in genes enriched for cognitive function

**Fig 3:**
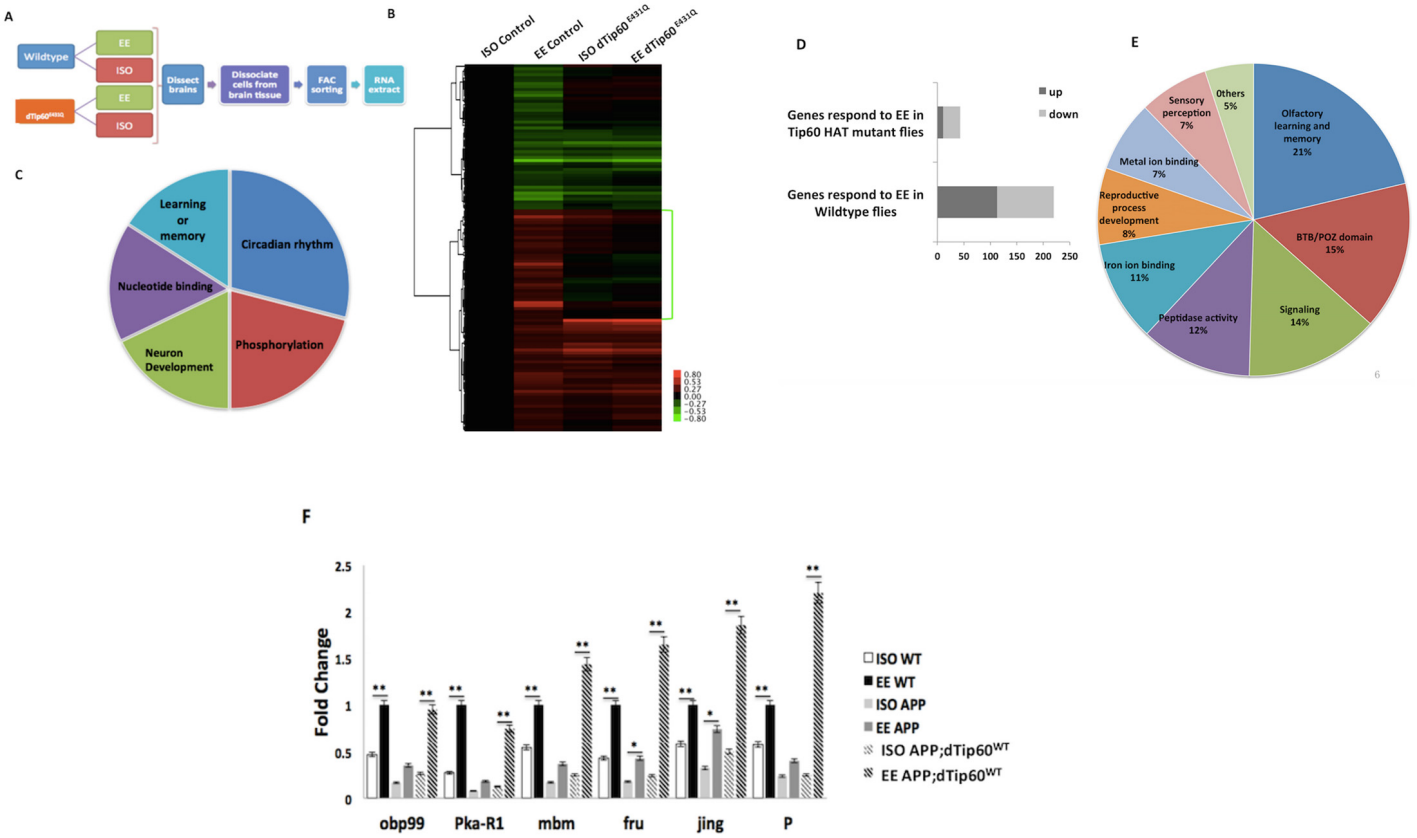
Tip60 promotes EE neuroadaptative transcriptional benefits under APP induced neurodegenerative conditions. (A) Experiment schematic for the microarray analysis. (B) The hierarchical cluster of the 220 genes differentially regulated in response to EE in the wild-type control flies (corrected P-value < 0.05, FC> 1.3) reveals an impaired transcriptional response in Tip60 HAT mutant flies. (C) Pie diagram showing GO term associated enrichment in each of the major function categories identified in the analysis. (D) Number of genes that are up-regulated (dark gray) and down-regulated (light gray) in response to EE in control and Tip60 HAT mutant fly MB neurons with FC>1.1. (E) Function analysis on the selected gene group (highlighted on heatmap). This subset of genes is not significantly misregulated in comparison to WT under ISO condition, and is not responsive to EE induced neuroadaptive transcriptional benefits. (F) Quantitative RT-PCR validation of representative gene targets in control, APP and APP;dTip60^WT^ flies under ISO and EE conditions for EE-mediated transcriptional regulation on the representative genes. qPCR was performed using RNA isolated from fly heads. Histogram represents fold change in gene expression using ΔΔCt method with RP49 as the internal control. Student t-test was used to determine statistical significance between different housing conditions within the same genotype. ** P < 0.01, *P < 0.05. Error bars indicate SEM.

EE has been shown to positively impact gene expression profiles in the mouse brain that are enriched in functions such as neuronal structure, synaptic plasticity and neurotransmission. Thus, we asked whether the EE induced beneficial MB structural and synaptic changes we observe are accompanied by neuroadaptive transcriptional benefits in the *Drosophila* MB, and if so, is Tip60 HAT action required for this process. To address this question, we crossed our UAS-mCD8-GFP;Tip60^E431Q^ flies or control UAS-mCD8-GFP flies to MB GAL4 OK-107 to simultaneously induce Tip60 HAT loss in the MB while tagging MB cells with GFP (Fig 3B). Adult progeny were exposed to EE or ISO conditions. After conditioning, the GFP tagged MB Kenyon neurons were FACs purified from conditioned fly brains from each genotype to enrich for detection of an EE induced MB transcriptional response. RNA was isolated from the purified Kenyon MB neurons and transcriptional changes for each genotype were assessed using microarray analysis (Fig 3A).

Bioinformatics comparative analysis of microarray data for EE versus ISO conditions for identical genotypic control wild type flies revealed significant differential expression for 220 genes, indicative of a neuroadaptative transcriptional response to EE (Fig 3B). Functional gene analysis revealed the top five enriched gene ontology (GO) groups that the EE responsive genes clustered to included processes known to be responsive to external stimuli such as circadian rhythm, neuron development and L&M, lending credibility to our analysis (Fig 3C). In contrast, when we directly compared EE versus ISO conditions for Tip60 HAT mutant flies, we found that only 43 genes exhibited differential expression, indicative of a lack of neuroadaptive response as a result of Tip60 HAT loss (Fig 3B and 3D).

Comparative analysis of microarray data from control w^1118^ flies versus Tip60 HAT mutant flies for EE data sets revealed transcriptional misregulation of 219 genes within the Tip60 HAT mutant flies. GO functional analysis on this subset of genes revealed that they are enriched for cellular processes such as methyltransferase activity, zinc protein finger DNA binding and nucleosomal organization. Of note, while these genes encode proteins known to function in epigenetic gene regulation, which is consistent with Tip60’s role in this process, these functions are not known to be directly responsive to external stimuli. In direct contrast, GO analysis of genes that are not misregulated in response to Tip60 HAT loss, but are impaired in their response to EE conditioning when compared to their ISO counterpart data sets, demonstrate that this subset of genes is enriched in processes known to be responsive to external stimuli that include olfactory learning and memory, sensory perception and signaling (Fig 3E). Taken together, these results suggest that Tip60 HAT action plays a role in mediating a beneficial activity dependent transcriptional neuroadaptive response to EE.

Our findings showed that EE induced beneficial MB structural and synaptic changes are compromised under APP neurodegenerative conditions and rescued by Tip60. As transcriptional regulation is a key mechanism by which HATs exert their action, we asked whether an EE neuroadaptative transcriptional response is compromised under APP neurodegenerative conditions and whether this impairment is relieved by increasing Tip60 HAT levels. To address this question, we selected six cognition associated EE responsive genes (Table 1) from our microarray analysis that did not display overall misregulation upon Tip60 HAT loss, but did display impairment in a transcriptional EE response when compared to their control w^1118^ counterparts. The genes we selected are as follows: Pka-R1 (cAMP-dependent protein kinase R1) is a direct Tip60 gene targets selected from our published ChIP-seq analysis[10] and its absence leads to olfactory learning defect that are observed in adult fly [55]. Mbm (mushroom body miniature) is essential in supporting the maintenance of MB Kenyon cell fibers in the larval stage and metamorphosis. Its loss leads to MB structural and cognitive function deficits[56] [57]. Fruitless (fru) mutant flies exhibit defects in axonal projections [58]. Jing, a zinc finger transcription factor, is involved in regulating neuronal and glial differentiation and survival in the developing brain [59]. Obp99a (Ordorant-binding protein 99a) is essential for olfactory perception of stimulus[60] and response to pheromone[61]. P (Pink) deficiency impairs synaptic function by blocking synaptic vesicle mobility during rapid neurotic stimulation[62]. We then carried out quantitative RT-PCR (qPCR) to assess their gene expression levels in EE versus ISO conditioned MB from control w^1118^, APP and APP;Tip60^WT^ fly genotypes. These qPCR results validated microarray results (Fig 3A) by demonstrating that the selected genes are upregulated in response to EE, and this EE response is compromised under APP neurodegenerative conditions in four out of the six genes tested and restored by Tip60 (Fig 3F). Taken together, these findings suggest that in adult fly MB neurons, a subset of genes undergo a beneficial neuroadaptive transcriptional response to EE and that Tip60 HAT activity plays a role in this process. Additionally, we show that this EE response is impaired under APP neurodegenerative conditions and rescued by Tip60, further implicating Tip60 HAT action in the EE response.

**Table 1:** Tip60 HAT activity is required to mediate EE induced neuroadaptive benefits.

Gene Symbol	Gene Name	Inferred Function
**Obp99a**	**Ordorant-binding protein 99a**	**Olfactory perception of stimulus and response to pheromone**
**Pka-R1**	**cAMP-dependent protein kinase R1**	**Olfactory learning**
**mbm**	**mushroom body miniature**	**Maintenance of MB Kenyon cell fibers in the larval stage and metamorphosis**
**fru**	**fruitless**	**Axonal projection**
**jing**	**jing**	**Neuronal and glial differentiation and survival in the developing brain**
**P**	**pink**	**Synaptic function by blocking synaptic vesicle mobility during rapid neurotic stimulation**

List of selected cognitive linked genes and their functions that are identified in Microarray analysis and validated in adult head tissue using quantitative RT-PCR.

### EE neuroadaptative transcriptional benefits in cognition associated gene expression involves Tip60 mediated histone acetylation induction at both promoter and gene-coding regions

EE mediated beneficial neuroadaptative changes have been shown to correlate with an induction of specific histone acetylation marks within the hippocampus and cortex regions of the mouse brain [41, 63, 64]. Our findings that increasing Tip60 HAT levels in the MB enhances EE neuroadaptative transcriptional benefits of cognition associated genes under APP neurodegenerative conditions prompted us to ask whether such expression changes are accompanied by histone acetylation induction. To test this, we crossed flies carrying a UAS-mCD8-GFP marker in conjunction with MB specific OK107-GAL4 driver to either control w^1118^, APP or Tip60^WT^;APP flies and assessed bulk levels of specific histone acetylation marks using western blotting of protein homogenates from EE or ISO conditioned adult whole fly brains for each genotype. We chose to measure the levels of histone H3 proteins acetylated at sites K9 and K14, and H4 proteins acetylated at sites K5, K12 and K16 as they are each associated with promoting cognition associated gene expression. Previously, H3K9, K14 and H4K5, K12 have been shown to be responsive to EE in the mouse brain [41]. Our results showed that control w^1118^ fly brains display a significant increase in H3K14ac, H4K5ac, H4K12ac and H4K16ac under EE conditions in comparison to ISO conditions. In direct contrast, APP flies only displayed a significant increase in H3K14ac in response to EE. We further found that increased Tip60 HAT levels under APP neurodegenerative conditions reinstates an EE induced induction response for histone acetylation marks H4K5ac and H4K12ac. Taken together, our results indicate that induction of H4K5 and H4K12 acetylation levels in response to EE requires Tip60 HAT action in APP neurodegenerative flies.

**Fig 4:**
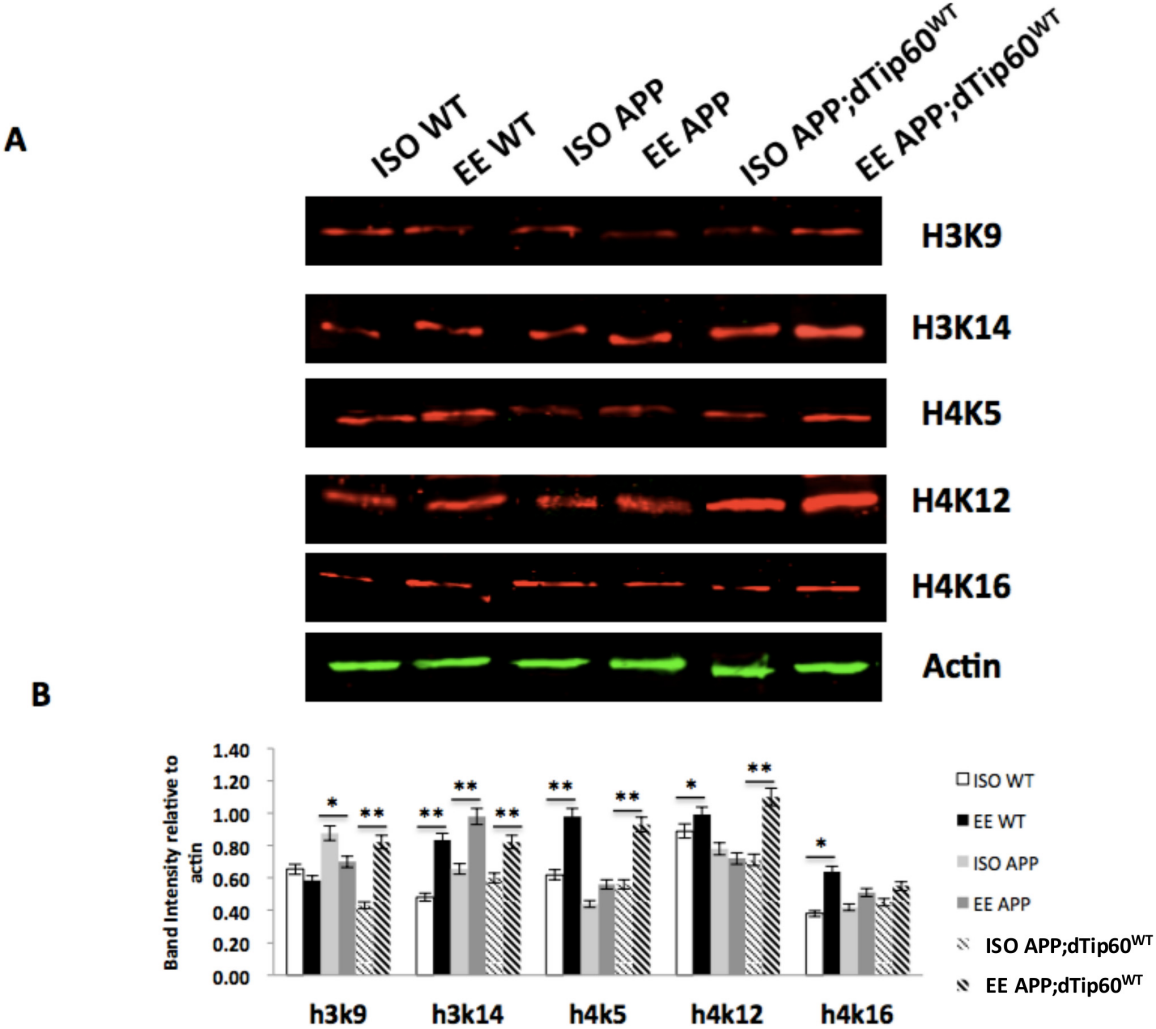
Restoration of H4K5 and H4K12 acetylation levels in response to EE requires Tip60 HAT action in APP flies. The indicated transgene was expressed in the fly MB using GFP;;OK107-Gal4 driver. Expression of human APP in MB results no significant acetylation change in response to EE, while increased Tip60 HAT activity restores such response in acetylation of H4K5 and H4K12. (A) Representative immunoblot showing histone acetylation in WT, APP and APP;dTip60^WT^ flies under ISO and EE conditions. (B) Quantification of (A). Independent (unpaired) Student t-test was used to determine statistical significance between EE versus ISO conditions within the same genotype for each histone acetyl marks. Each blot was repeated at least three times. Each histone sample was extracted from fly heads. Proteins were extracted from at least two independent pooled tissue samples (30 heads for each extract). Each sample was run at least three times for statistic analysis. ** P < 0.01, *P < 0.05. Error bars indicate SEM.

**Fig 5:**
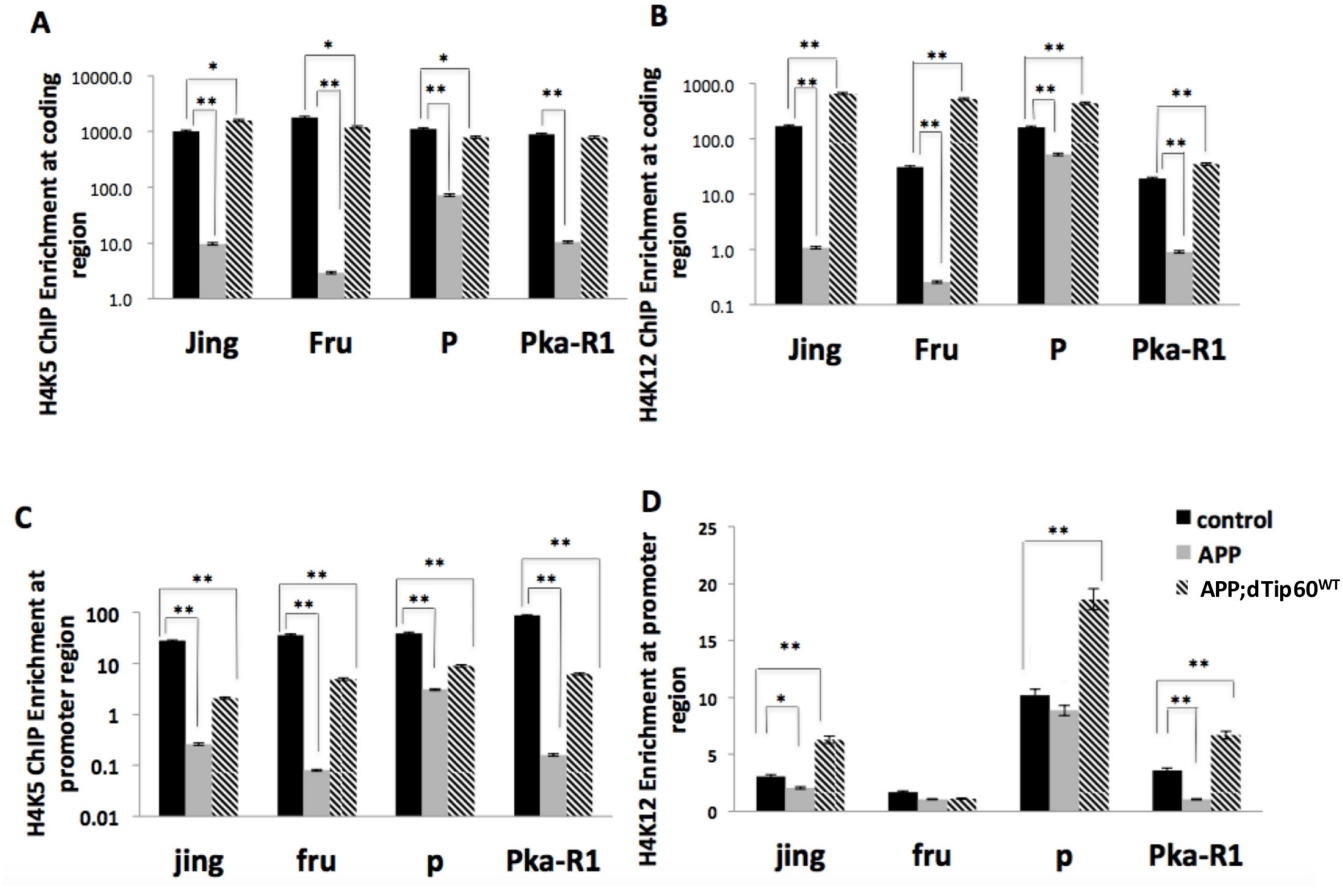
Tip60 HAT activity restores reduced histone acetylation at both promoter and gene coding regions of EE responsive genes under APP neurodegenerative conditions. H4K5ac (A) and H4K12ac (B) enrichment at gene coding region of selected EE responsive genes. H4K5ac (C) and H4K12ac (D) enrichment at the promoter region of same EE responsive genes. Enhanced acetylation levels were observed in wild-type flies, while such EE induced response was reduced in APP flies, and restored with excess Tip60. Excess Tip60 increased EE-induced H4K5 and H4K12 acetylation at both promoter and gene coding regions of selected EE responsive genes (A)H4K5ac ChIP of coding region. (B)H4K12ac ChIP of coding region. (C)H4K5ac ChIP of selected promoter region. (D)H4K12ac ChIP of selected promoter region. Newly eclosed adult fly progeny were exposed to a group of 30 flies (1:1 sex ratio) to generate EE conditions as previously described. Fold enrichment was compared among control, APP and APP;dTip60^WT^ flies as follows: one-way ANOVA analysis was used to determine the probability that there were differences between the variants. In the cases that ANOVA indicated that there was a significant difference between variants (p<0.05), we performed post hoc pair-wise comparisons using the Bonferroni correction to determine the significance between each genotype. ** P < 0.01, *P < 0.05. Fly heads (n >1000) were collected for ChIP experiments. Error bars indicate SEM.

Regulation of acetylation levels on specific histone lysine residues at distinct gene loci is a key mechanism by which Tip60 exerts epigenetic control over transcriptional activity. Thus, we asked whether the Tip60 mediated EE increase in bulk histone acetylation we observed (Fig 4A) affects the acetylation status of the promoter and gene-coding regions at some of the EE responsive genes we identified in the MB. We further asked whether this response is compromised under APP neurodegenerative conditions and restored by an increase of Tip60 HAT levels. To address these questions, we performed chromatin immunoprecipitation (ChIP-qPCR) on chromatin isolated from adult fly heads from control w^1118^, APP and APP;Tip60^WT^ fly genotypes to assess acetylation levels. We chose EE responsive histone H4K5 and H4K12 marks at select EE responsive genes (Jing, fru, P, Pka-R1; Fig 3F) we identified by MB FACs/microarray analysis. To understand how acetylation regulates activity-dependent gene transcription, we chose two different loci to assess acetylation levels at these gene targets. For the gene-coding region, we chose a site 1kb downstream of the transcriptional start site (TSS), which marks the beginning of the transcriptional initiation. To identify a potential Tip60 binding site at the promoter region, we focused on a genomic site 1kb up-stream of the TSS (S2A Fig). As Tip60 is thought to be recruited to gene promoters *via* promoter bound transcription factors (TFs), we focused on first identifying TF binding consensus sequences among our activity-dependent gene targets using bioinformatics tool MEME-ChIP. Within this region, we found a specific consensus site for the TF br (broad), known to play an important role in dendritic morphogenesis and CNS development in fly nervous system[65]. Therefore, we chose this specific region to assess promoter acetylation enrichment (S2A and S2B Fig). In control w^1118^ flies, we found enrichment for H4k5ac and H4K12ac at both the promoter and gene-coding regions for each of the selected EE responsive cognition associated genes, supporting a role of H4K5ac and H4K12ac in their transcriptional activation (Fig 5). Further, consistent with our prior transcriptional analysis of these genes (Fig 3F), we observed a significant reduction of histone acetylation at H4K5 and H4K12 marks under APP neurodegenerative conditions in APP flies that was restored by increasing Tip60 HAT levels. Of note, when comparing acetylation enrichment levels between gene-coding and promoter regions, we observed that enrichment levels at the promoter sites were significantly lower than at the gene-coding region, suggesting different acetylation based mechanisms of action at these discrete promoter versus gene-coding regions.

**Fig 6:**
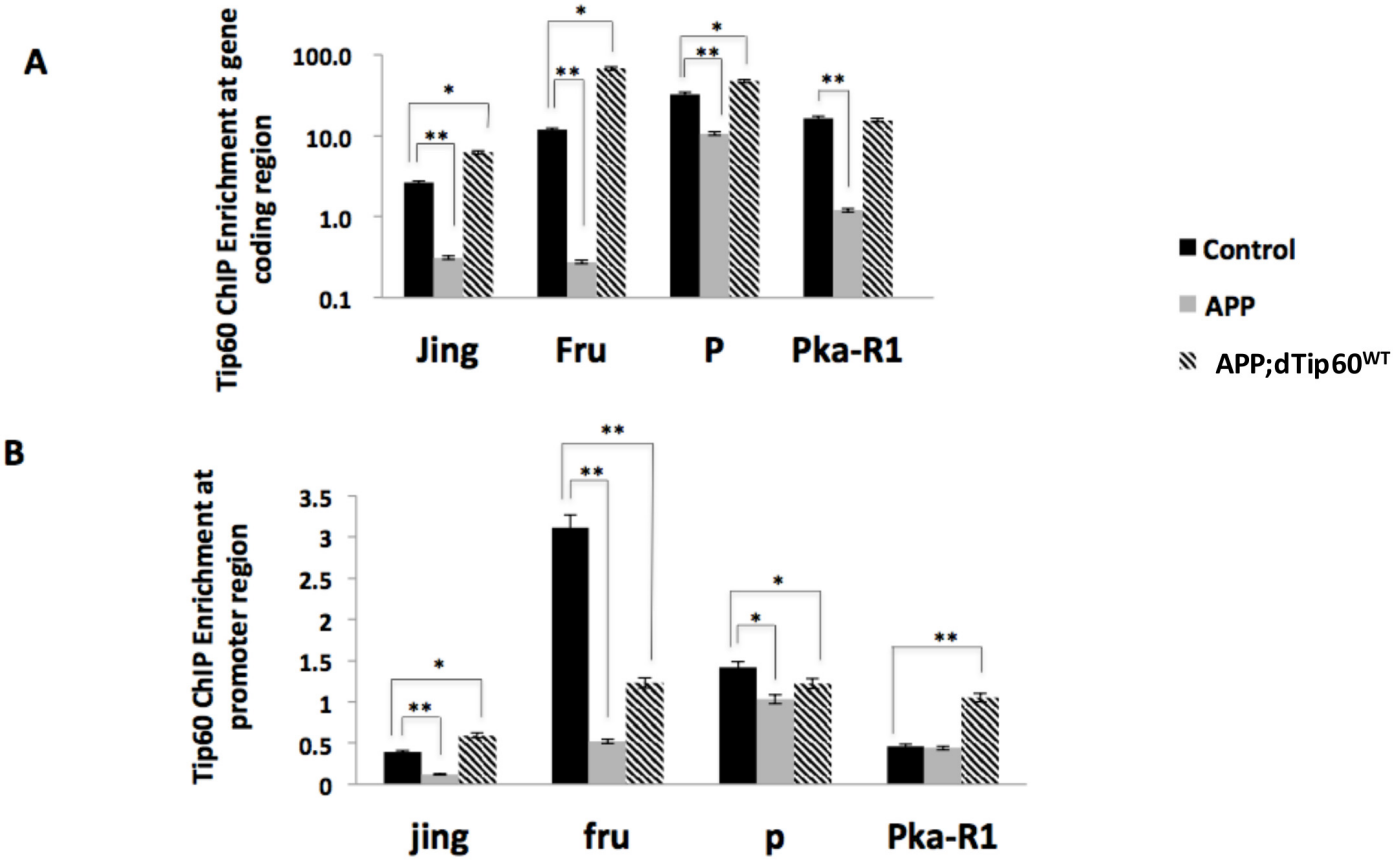
Tip60 restores reduced histone acetylation levels under EE conditions by directly binding at both promoter and gene coding regions of cognition gene loci under APP induced neurodegenerative conditions. Tip60 displays similar enrichment pattern as histone acetylation on wild-type, APP flies and APP;Tip60 fly. (A)Tip60 ChIP at coding region. (B)Tip60 ChIP at selected promoter region. Newly enclosed adult fly progeny were exposed to a group of 30 flies (1:1 sex ratio) to generate EE conditions as previously described. Fold enrichment was compared among control, APP and APP;dTip60^WT^ flies as follows: one-way ANOVA analysis was used to determine the probability that there were differences between the variants. In the cases that ANOVA indicated that there was a significant difference between variants (p<0.05), post hoc pair-wise comparisons were then performed using the Bonferroni correction, to test the significance between genotypes. ** P < 0.01, *P < 0.05. Fly heads (n >1000) were collected for ChIP experiments. Error bars indicate SEM.

Given our finding that histone acetylation enrichment at EE responsive genes was significantly reduced under APP neurodegenerative conditions and restored by Tip60, we speculated that the acetylation reduction we observed in APP flies was caused by impairment of recruitment of Tip60 to both promoter and gene-coding regions. To test this, we again carried out ChIP, this time to assess Tip60 enrichment at the EE responsive gene-coding and promoter regions using chromatin isolated from adult fly heads of control w^1118^, APP and APP;Tip60^WT^ genotypes. Similar to the histone acetylation enrichment pattern we observed in these genotypes (Fig 5A–5D), we found that with the exception of gene Pka-R1, levels of Tip60 were enriched in control w^1118^ flies, significantly reduced in APP flies, and restored in APP;dTip60^WT^ flies under EE conditions. Interestingly, enrichment levels for Tip60 were significantly higher within the gene-coding region when compared with the promoter region (Fig 6A), similar to what we observed for histone acetylation enrichment at H4K5 and H4K12 (Fig 5A and 5B). These findings suggest that Tip60 mediates histone H4K5 and H4K12 acetylation at both the promoter and within the gene-coding region under EE conditions to regulate activity-dependent gene expression and that mechanisms underlying this mode of transcriptional control might differ between these two sites.

### Extracellular stimulation of rat hippocampal neurons induces nuclear import of Tip60

**Fig 7:**
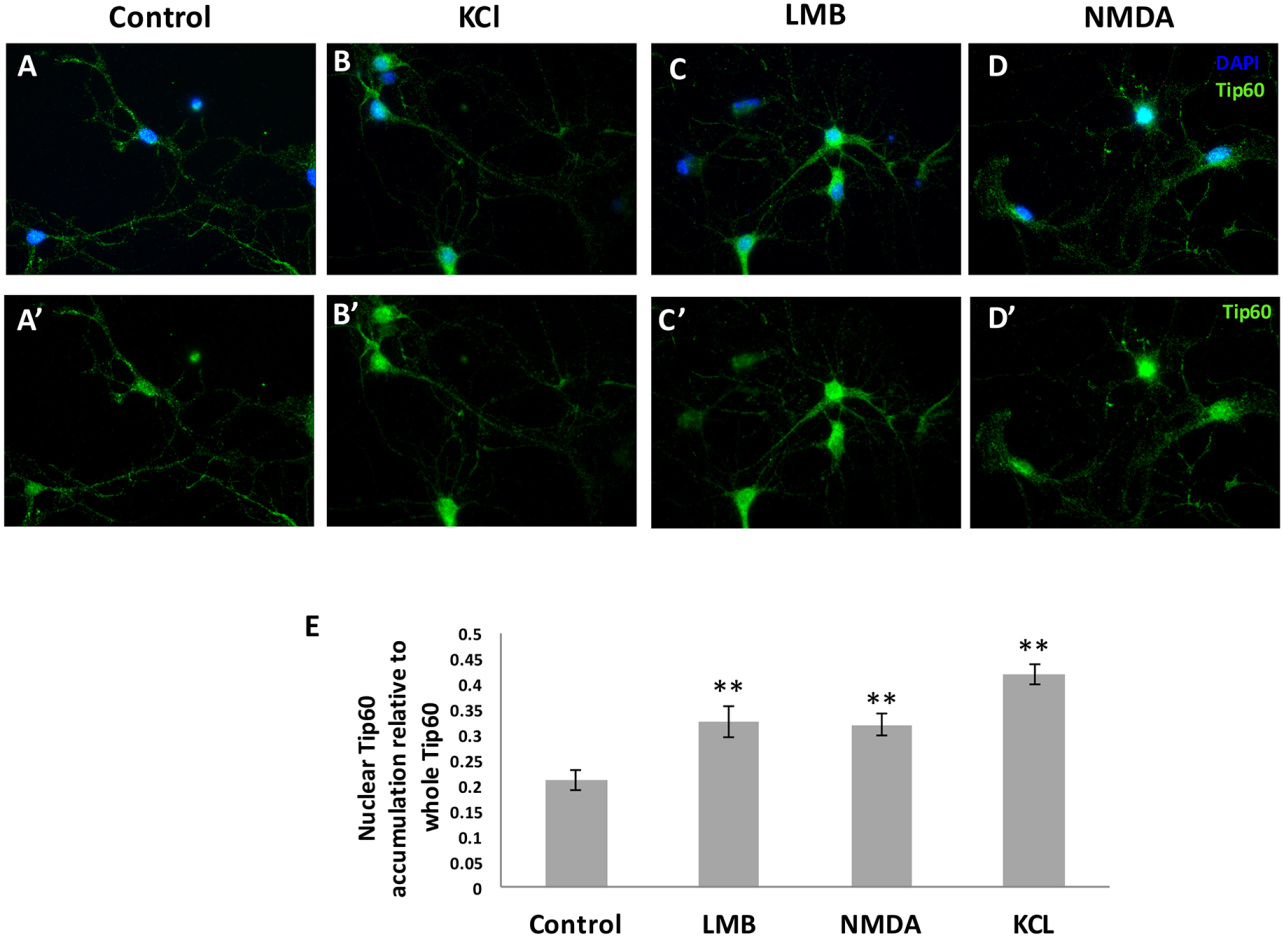
Extracellular stimulation of hippocampal neurons induces Tip60 nuclear import. (A, A’) Tip60 distribution in nucleus and cytoplasm under resting conditions. (B-D; B’-D’) Rat Hippocampal neurons (DIV6) treated with depolarizing agent NMDA immunostained with Abs to Tip60, Tau(cytoplasm) and DAPI(nucleus). (E)Quantification of fluorescent signal intensity for nuclear Tip60 in neurons treated with Leptomycin B(LMB) that blocks nuclear export, or depolarizing agents NMDA or KCl. Neurons (n = 50) were analyzed per treatment. Data represents the mean of 50 replicates. One-way ANOVA was used to determine statistical significance between control and each of treatment conditions. ** P < 0.01, *P < 0.05. Error bars indicate SEM.

We next sought to investigate a potential mechanism for how Tip60 carries out its transcriptional role in the nucleus in response to external stimuli. Studies have shown that neural activity modulates chromatin acetylation by influencing certain HDACs to shuttle in and out of the nucleus[66, 67]. We previously reported that Tip60 displays a nuclear-cytoplasmic distribution in fly neural circuits such as the MB [10] and neural muscular junction (NMJ) [8]. Consistent with our findings, Tip60 also contains both a nuclear localization signal (NLS) and nuclear export signal (NES)[18]. Thus, we asked whether Tip60 has nuclear-cytoplasmic shuttling capabilities, and whether neuronal activity would modulate such Tip60 cellular distribution. To address this question, we chose to utilize primary rat hippocampal neurons as these neurons are substantially larger than the fly MB Kenyon cells, thus enabling us to carry out higher resolution analysis on Tip60 cellular distribution in response to external stimulus. Further, we wished to confirm that the Tip60 nuclear/cytoplasmic cellular distribution pattern we observed in fly neural circuits is conserved in the mammalian brain. To address these questions, we first used immunohistochemistry staining with antibodies against Tip60, cytoplasmic marker MAP2 and nuclear neuronal marker DAPI to visualize Tip60 distribution in hippocampal neurons of day *in vitro* (DIV) 6 (Fig 7A–7C). Our data revealed a nuclear and cytoplasmic distribution pattern for Tip60 in primary rat hippocampal neurons (S3 Fig), similar to what we observe in the fly neuronal circuits[8].

We next asked whether Tip60 shuttles between the nucleus and cytoplasm. To address this question, we tested whether blocking nuclear export of proteins by leptomycin B (LMB) in primary rat hippocampal neurons results in an increase in Tip60 nuclear accumulation. LMB blocks chromosome region maintenance 1 (CRM1) that mediates nuclear export. Our results demonstrate that Tip60 is retained in the nucleus upon exposure to LMB, indicating that Tip60 undergoes continuous shuttling between nuclear-cytoplasmic compartments in the rat hippocampal neuronal cells. To ask whether this shuttling mechanism responds to external stimuli, we stimulated the rat hippocampal DIV6 neurons with either NMDA or KCl for 30 minutes and assessed the cellular distribution of Tip60 using immunohistochemistry staining with antibodies to Tip60 and nuclear marker DAPI (Fig 7A–7C, 7A’–7C’). We selected NMDA because it is a well-characterized glutamate receptor agonist involved in learning and memory function[68] and *Drosophila* has NMDA receptor homologs (dNR1,dNR2,dNR3) that have been implicated in MB function in learning and memory [69]. We also chose to use KCl as its depolarization triggers presynaptic release of glutamate and subsequently activates postsynaptic glutamate receptors[70] involved in learning and memory. After a thirty-minute treatment, neurons were fixed and subjected to immunochemistry staining (Fig 7A–7D and 7A’–7D’). Quantification on Tip60 distribution revealed a significantly stronger Tip60 signal in the nucleus (Fig 7E), suggesting activation of NMDARs induced Tip60 shuttling into the nucleus. Together, our findings suggest that Tip60 responds to extracellular cues *via* its nuclear import to mediate nuclear epigenetic control of activity-dependent cognition associated gene expression profiles.

## Discussion

In the present study, we investigate whether EE mediates neuroadaptative benefits by promoting Tip60 HAT action in cognition linked gene control. Using a well-established social EE paradigm in *Drosophila* we show that EE conditions induce significant beneficial structural changes that include axonal outgrowth in the mushroom body (MB) of control w^1118^ flies when compared to their genetically identical siblings housed in isolated (ISO) conditions. Further, we find that loss or gain of Tip60 HAT levels specifically in the MB results in a loss of this beneficial EE mediated MB axonal outgrowth response, indicating that appropriate levels of Tip60 are required for EE neuroadaptative structural MB benefits. Consistent with our findings, elegant work has shown that CBP HAT deficient mice are also impaired in responding to positive EE effects that include enhancement of hippocampal neurogenesis, synaptic transmission and promoting histone acetylation dependent transcription of CBP target genes required for these processes, supporting a critical role for CBP as a mediator of EE-induced benefits[32]. Thus, our findings add Tip60 to the small repertoire of cognition linked specific HATs shown to respond to external cues.

Increasing compelling evidence supports the premise that the severity of AD pathology can be influenced by a complex interplay between genetic and environmental risk factors that can either slow or exacerbate AD progression. Accordingly, EE conditions comprising positive social reinforcements are beneficial for reinstating long-term cognitive ability in neuropathological conditions such as AD even after significant brain impairment, including atrophy, has occurred[20–23, 32]. For example, Dong et al show that EE conditions restore long-term learning and memory function in a mouse model for neurodegeneration that has undergone synaptic and neuronal loss by promoting hippocampal neurogenesis, dendrite sprouting and synaptic connections [22, 32]. In direct contrast, stressful isolation conditions exacerbated neurodegenerative pathology [71]. Here we show that while positive EE conditions do not significantly alleviate APP induced MB morphological defects in the fly, EE conditions do induce positive changes that include an induction of synaptic vesicle protein markers CSP and SYX in the fly brain. Of note, Jankowsky et al showed that EE mitigates cognitive deficits in two types of AD mouse models using a complex social EE paradigm involving social interaction in combination with novel objects, nesting materials and exercise wheels. Interestingly, the AD mouse model overproducing amyloid–β used in this study exhibited less significant EE induced neuroadaptative benefits when compared to an APP Swedish mutation AD mouse model. These data, in conjunction with our findings here, indicate that different EE paradigms and AD models in varying species can elicit varied but still beneficial positive effects. Interestingly, we show that both loss and gain of Tip60 HAT levels in the fly MB result in a reduction of MB outgrowth and lack of response to EE in 5 day old flies, suggesting that appropriate levels of Tip60 HAT activity are required for general MB morphology as well as EE mediated neuroadaptative morphological benefits. Of note, the reduction in MB outgrowth in response to excess Tip60 is in contrast to our previous studies demonstrating that in 2–3 day old adult flies excess Tip60 leads to no observable detrimental effect on MB morphology [2, 5–7, 10]. We speculate that this difference is likely due to the older adult flies used here as well as the distinct EE and ISO housing conditions utilized in the present study versus the standard housing conditions we used in our previous study in which hundreds of flies are housed together in large bottles. Remarkably, we found that increasing Tip60 HAT levels specifically in the MB, fully restored a beneficial EE response under APP neurodegenerative conditions in terms of enhancing axonal outgrowth and restoring induction of DLG and BRP synaptic markers. Based on our findings, we speculate that Tip60 serves as a positive mediator in translating external EE conditions into positive hard wiring changes in the brain and that this function is compromised under APP neurodegenerative conditions.

During development and in adult animals, neurons in the brain respond to changes in environment in large part *via* changes in gene expression. One of the most critical experience-driven behavioral change is learning and memory formation, as it directly impacts cognitive ability [20, 72–74] and numerous studies support a critical role for the transcription of DNA in the memory formation process[75]. Indeed, mice raised under EE conditions show changes in the expression of genes in the brain involved in formation of new synapses, strengthening of existing synapses, neurotransmission as well as cytoskeletal changes involved in promoting neurogenesis[76]. Specific HATs play a key role in epigenetic regulation of gene expression profiles essential for maintaining neuronal health and mediating higher order brain functions[3, 4, 66, 77–79]. These studies support the premise that HATs may also play a role in mediating EE transcriptional control in learning and memory. Although the full repertoire of cognition-linked HATs that respond to external cues remains largely unknown, elegant work by Kim et al [80] support a critical role for CBP as a mediator of EE-induced neurotranscriptional benefits. These studies reveal that CBP utilizes distinct activity-dependent receptors and Ca2+ signaling pathways to link its action in triggering plasticity associated gene transcription profiles in response to external cues[81]. Accordingly, our bioinformatics analysis of microarray data from FACs sorted MB Kenyon cells in EE vs. ISO conditioned wild-type control flies revealed significant differential expression for 220 genes, indicative of a neuroadaptative transcriptional response to EE. Functional analysis revealed the top 5 enriched gene ontology (GO) groups that the EE responsive genes clustered to included processes known to be responsive to external stimuli such as circadian rhythm, neuron development and L&M, lending credibility to our analysis. In direct contrast, only 43 EE responsive genes revealed differential expression in Tip60 HAT mutant MB, indicative of impairment of the neuroadaptative response as a result of Tip60 HAT loss (Fig 3D). GO analysis of genes that are not misregulated in response to Tip60 HAT loss, but are impaired in an EE response reveal that this subset of genes is enriched in processes known to be responsive to external stimuli that include olfactory learning and memory, sensory perception and signaling (Fig 3E). Based on these findings, we speculate that Tip60 HAT action plays a role in mediating a beneficial activity dependent transcriptional neuroadaptive response to EE to promote beneficial MB morphological changes and function. Of note, our microarray analysis also reveals a fraction of genes that are misregulated in the Tip60 HAT mutant MB that function in general neural development. Moreover, while we observe no defects in third instar larval MB in response to loss or gain of Tip60, we do observe axonal outgrowth defects in the Tip60 HAT mutant adult MB. Therefore, we do not rule out the possibility that loss of Tip60 also causes transcriptional defects in genes required for general adult MB development, thereby contributing to the compromising MB response to EE we observe.

Dynamic epigenetic regulation of activity-dependent neuronal gene expression profiles is emerging as a fundamental mechanism by which neurons adapt and fine-tune their transcriptional responses to environmental cues to promote sustained neural plasticity and higher order brain function[3, 21, 66, 77]. In support of this concept, Fischer et al demonstrated that EE conditions trigger hippocampal induction of histone acetylation specifically at marks H3 (K9, K14) and H4 (K5, K8, K12) [22]. Our analysis of EE impacted histone acetylation marks in the fly MB show remarkable similarity to these mouse studies in that control w^1118^ fly brains display a significant increase in H3K14ac, H4K5ac, H4K12ac under EE condition, suggesting that the EE induced histone acetylation induction response is tightly conserved in both flies and mouse. Of note, we also observed a significant induction in H4K16ac in response to EE in the fly MB that was not shown in mice, suggesting that different EE paradigms and species can elicit varied but still beneficial positive effects. Consistent with numerous studies showing decreased histone acetylation levels in the brain a variety of mouse AD models [82, 83] and in the temporal lobes of human patients with AD [84, 85], here we show that the EE histone acetylation induction response was dampened in APP neurodegenerative fly brains. Remarkably, increasing Tip60 HAT levels in the APP neurodegenerative MB brain restored an EE induction response for histone H4K5ac and H4K12ac marks. These results suggest that Tip60 HAT action is important in the EE histone acetylation induction response and that histone H4K5ac and H4K12ac are putative EE mediated Tip60 HAT targets. Further, ChIP analysis of 4 cognition linked genes impaired in EE response in the Tip60 HAT mutant MB revealed enrichment for Tip60 and histone acetylation at these putative Tip60 histone lysine targets. Notably, we found that enrichment levels for Tip60 and histone acetylation at marks H4K5 and H4K12 were significantly higher within the gene-coding region when compared with the promoter region. Our findings are not unprecedented as Fischer et al[22]showed in mouse brain that acetylation at mark H4K12 is selectively associated with the coding regions of genes normally transcriptionally activated during the learning processes [20, 22, 32] and was not observed for other genomic regions including the transcriptional start site (TSS). Moreover, such H4K12 gene coding region acetylation spread was impaired in the aged mouse brain with concomitant disruption of transcriptional activation. Other groups have also shown gene coding enrichment for certain HATs and their acetylation profiles in various model organisms. For example, Johnsson et al[86] show in yeast that the HAT Gcn5 is predominantly localized to the coding regions of highly transcripted genes to modulate H3K14ac levels and transcriptional elongation in response to environmental conditions such as stress. Moreover, the human Elongator complex that contains the HAT Elp3 was shown to be essential for neuronal function[87] and was found to complex with RNA pol II to facilitate transcription through chromatin in a acetyl-CoA dependent fashion. Based on these data, we speculate that Tip60 functions to mediate activity dependent gene expression by promoting gene coding histone acetylation spread to maintain genes in a state well poised for rapid and robust transcriptional induction in response to changing external cues, and that such Tip60 mechanisms are compromised in the neurodegenerative brain.

An important question to consider is how does Tip60 respond to external environmental stimuli to mediate a transcriptional response in neurons? Neural activity has been shown to modulate chromatin acetylation in hippocampal neurons in part, by controlling shuttling of certain HDACs in and out of the nucleus that influence their activity in gene control[66, 67]. Intriguingly, we observe both cytoplasmic and nuclear localization for Tip60 in activity dependent fly neuronal circuits that include the NMJ synaptic boutons and MB Kenyon cells[8, 10]. Additionally, Tip60 contains a nuclear localization (NLS) and nuclear export (NES) sequence that we predicted might mediate its shuttling between nucleus and cytoplasm[18]. Accordingly, here we are the first to show a cytoplasmic and nuclear distribution pattern for Tip60 in primary rat hippocampal neurons and that treatment of these neurons with depolarization inducing extracellular factors promote uptake of Tip60 into the nucleus (Fig 3C–3E). Based on these findings, we propose a model by which external stimuli that is read as synaptic input induces Tip60 shuttling into the nucleus that in turn, influences the neural epigenetic acetylation landscape and gene activity.

The histone acetylation status of chromatin and concomitant activity dependent gene control in the brain have been shown to become impaired during the lifetime of a neuron via mechanisms involving loss of certain HAT function and a decrease in histone acetylation[88–92]. Accordingly, these changes are tightly linked to a variety of age related neurological disorders[3, 4, 41, 84, 85, 93] that include Parkinson’s, Alzheimer’s [2, 7, 64] and Huntington’s diseases[94]. Moreover, epigenetic misregulation of activity dependent genes is also tightly linked to early developmental neurological disorders such as autism and Rubinstein Taybi syndrome[95]. Cognitive behavioral intervention approaches are gaining credibility as important non-invasive ways to slow the progression of age related neurodegenerative disorders [96, 97] as well as to reverse cognitive deficits in early childhood disorders such as autism[98]. In support of these studies, here we show that EE conditions provides some beneficial changes in the APP neurodegenerative fly brain, and that these positive changes can be significantly enhanced by increasing Tip60 HAT levels. Accordingly, pharmacological treatments aimed at increasing global acetylation levels through the use of non-selective pan-HDAC inhibitors have shown promising effects in reversing cognitive deficits in a variety of neurodegenerative animal models [99] making this a powerful therapeutic strategy [100–103]. However, many currently used HDAC inhibitors (HDACi) lack target specificity[2, 104–109] and act by increasing global acetylation levels in the brain with potential detrimental effects, raising concerns about their applicability. Unlike some HDACs [105, 106], select HATs like Tip60 have non-redundant neural functions that may not only restore general acetylation balance, but also modulate particular gene expression programs that work together to promote neuronal health. Thus, our findings implicate Tip60 as a critical mediator of EE-induced benefits and provide new insights into HAT based drug design that could compliment non-invasive behavioral strategies for early therapeutic intervention of cognitive disorders.

## Materials and Methods

### Fly stocks and maintenance

Flies were reared on standard medium (cornmeal/sugar/ yeast) at 25°C with a 12-hr light/dark (LD) cycle. W^1118^ flies were used as wild-type controls. OK107-GAL4 and UAS- GFP stocks were obtained from the Bloomington Drosophila Stock Center (Indiana University). UAS-CD8::GFP;; OK107-GAL4 stock was gifted by Dr. Steven Robinow from University of Hawaii. The generation and characterization of UAS-dTip60^E431Q^ and UAS-dTip60^WT^ flies are described in Lorbeck et al[1]. Generation and characterization of the double-transgenic UAS-APP;Tip60^WT^ fly lines are described in Pirooznia et al. All of the UAS-dTip60 fly lines described here are contained within a w^1118^ genetic background. Additionally, for all experiments, transgene expression levels for each of the UAS fly lines were assessed as described (Lorbeck et al. 2011; Pirooznia et al. 2012a,b; Johnson et al. 2013) to ensure that the different transgenic lines used for phenotype comparisons show equivalent dTip60E431Q, dTip60WT, and APP expression levels.

### Immunohistochemistry and antibodies

Adult *Drosophila* brains were dissected in PBS, fixed in 4% paraformaldehyde in PBS, washed three times in PBS containing 0.1% Triton X-100, blocked for 1 hr at room temperature (RT) in PBT containing 5% normal goat serum, and incubated with primary antibodies in blocking solution overnight at 4°C. Anti-Tip60 (1:400) was obtained from Abcam, anti-Fasciclin (mAb1D4; 1:10) was obtained from the Developmental Studies Hybridoma Bank (University of Iowa). Anti-GFP (1:100) was obtained from Millipore. Samples were washed three times in PBST at RT, and secondary antibodies (Jackson Immunoresearch) were applied in blocking solution for 2 hr at RT. After washing three times in PBS, samples were mounted in Vectashield (Vector Laboratories).

### Imaging and quantification

Adult brain preparations were imaged using the appropriate secondary antibodies. Anti-GFP and anti-Fasciclin immunostaining were visualized using Alexa-Fluor 488 and Alexa-Fluor 568. Confocal microscopy was performed using an Olympus microscope with fluoview acquisition software (Olympus, Center Valley, PA). Images were displayed as projections of 1-mM serial Z-sections. Consecutive subsets of the Z-stacks were utilized for the final projection. Images were adjusted for brightness and contrast using the ImageJ program to more clearly define MBs. Area of the MB lobes in the different genotypes was measured using National Institutes of Health ImageJ software.

### Quantitative western blotting

All western blots were carried on protein extracts from dissected entire heads. Flies from each housing condition were dissected and homogenized in groups of 30. All antibodies were obtained from the Developmental Studies Hybridoma Bank (University of Iowa): nc82 (a-BRP) 1:1000; 4F3 (a-DLG) 1:2500; 1G12 (a-CSP) 1:1000; 8C3 (a-Syx1A) 1:1000; JLA20 (β-actin) 1:300.

Histones were extracted from 50 fly heads from each genotypes and housing conditions using the EpiQuik^™^ total histone extraction kit (Epigentek Group Inc.) according to the manufacturer's protocol. Equal amounts of protein as quantitated by using a BCA protein assay kit (Thermo Scientific) were loaded onto a 16% SDS PAGE gel (29:1 acrylamide/bisacrylamide). Protein samples were denatured at 95°C for 15 min prior to loading. The fractionated proteins were electro-blotted onto nitrocellulose membrane (Biorad). The membrane was blocked with 3% BSA for 2 h at room temperature and then incubated overnight at 4°C with antibodies (Active motif) that recognizes four acetylated lysine residues (H3K9, H3K14, H4K5, H4K12 and H4K16) of histone H3 and H4. The membrane was washed three times with 0.1% TBST (50 mM Tris-Hcl (pH 7.4), 150 mM NaCl, 0.3% Tween 20) and incubated with secondary antibody for 1 h at room temperature. The membrane was washed three times with 0.1% TBST.

Western detection was done using chemiluminiscence (ECL kit, Thermo Scientific). Signals were quantitated using a Fluorchem imager (Alpha Innotech). To ensure signals were in the linear range, Alpha Ease FC software (Alpha Innotech, San Leandro, CA) was used according to the manufacturer’s instructions to select exposure times such that there was no saturation detected. Western analysis was repeated three separate times with two independent tissue extractions. Western blots and quantifications were performed as previously described[1]. Specifically, ECL signal intensities were quantified using ImageJ and normalized by dividing the within-lane actin signal (used as loading control). Statistical analysis was done fitting the normalized protein/actin ratios using Student's t-test for two-groups comparisons.

### Quantitative RT–PCR and ChIP assays

Total RNA was isolated from adult fly heads using the RNeasy Plus Mini Kit (QIAGEN). Complementary DNA (cDNA) was prepared using the SuperScript II reverse transcriptase kit (Invitrogen) according to the manufacturer’s instructions with 1 mg total RNA and 0.2 mg/ml random hexamer primers (Roche Applied Science). PCRs were performed in a 20ul reaction volume containing cDNA, 1 mM Power SYBR Green PCR Master Mix (Applied Biosystems), and 10 mM of both forward and reverse primers (primer pairs available upon request). PCR was performed using an ABI 7500 Real-Time PCR system (Applied Biosystems) following the manufacturer’s instructions. Fold change in messenger RNA expression was determined by the ΔΔCt method.

Chromatin precipitation assays were performed using truChIP^TM^ Chromatin Shearing Kit from Covaris, following the manufacturer’s instructions. Briefly, chromatin immunoprecipitation (ChIP) was carried out with 50 mg of sheared chromatin using three different antibodies: (i) 1 ug of RNA Pol II antibody (Abcam, Cambridge, MA); (ii) 1 ug of Tip60 antibody (Abcam); and (iii) 1ug H4K5ac and H4K12ac antibodies (Active motif). A mock reaction containing all reagents except the antibody was also set up as a control. The chromatin was immunoprecipitated using the EZ-Magna ChIP^™^ A—Chromatin Immunoprecipitation Kit (Millipore) exactly following the manufacturer’s specifications. The eluted material from the immunoprecipitation was then purified using a QIAquick PCR purification kit (Qiagen) and was directly used for real-time PCR. Primer sets designed by NCBI/ Primer-BLAST (www.ncbi.nlm.nih.gov/tools/primer-blast/). Primers are available upon request.

### Dissociation of Brain Neurons and FACS Analysis

Homozygous females from the Gal4 enhancer trap line OK107 were crossed to homozygous males from the reporter strain GFP, and GFP;dTip60^E431Q^. Approximately 100 male and female brains from the progeny of this cross were dissected and dissociated. The brains were removed from the head capsule with two fine-tip forceps and placed in Schneider’s medium (IM-009-B, Specialty Media) without serum. The medium was carefully removed, with enough left behind to cover the brains. Five hundred microliters of D-PBS without Ca^2+^ and Mg^2+^ (BSS-1006, Specialty Media) was added to gently wash the tissue, and the medium was removed again. This wash was repeated twice. After the last wash, 200 ml D-PBS without Ca^2+^and Mg^2+^ (room temperature) and 200 ml Low Trypsin–High EDTA solution (SM- 2004-C, Specialty Media) were added, and the solution was mixed gently with a micropipette. After 2–3 min, the brains were sucked into the pipette with FluoroPel pipette tips (Ulster Scientific), first just to loosen the tissue and then to dissociate the brains gradually into single cells or small cell clusters. Two hundred microliters of Schneider’s medium with 10% FBS was added, and the cell suspension was then transferred into a 1.5 ml microcentrifuge tube The dish was rinsed twice with 500 ml Schneider’s medium without serum to recover remaining cells. The tube was kept on ice for a few minutes to allow the clearly visible pieces of tissue to settle to the bottom of the tube. The cell suspension was then transferred into a new tube and used for FACS analysis. GFP-positive cells were sorted in a flow cytometer by monitoring.

### Microarray analysis

Total RNA was extracted from the sorted MB neurons and subjected to microarray analysis. RNA samples were hybridized to GeneChips Drosophila Gene 2.0 Arrays according to the manufacturer’s protocol (Affymetrix, Santa Clara, CA). ISO conditions for control flies serve as expression level baseline. The microarray data were then analyzed using R. RMA (Robust Multichip Average) algorithm was used for data normalization. Correlation matrix analysis was also performed using R, validating significant consistency of the microarray data for each of the three genotypes analyzed. Student T-test function was used to identify genes whose expression differed significantly (p<0.05) and these genes were then filtered to select for those that showed a 1.3-fold or greater change and a 95% confidence bound of fold change. Genes were annotated and biological processes were analyzed using the Database for Annotation, Visualization, and Integrated Discovery (DAVID) (http://www.david.abcc.ncifcrf.gov) [110, 111]. Promoter and gene coding region sequence was extracted from UCSC genome browser. Meme-ChIP was used for transcription factor binding site discovery [112].

### Cell culture

Cultures of rat hippocampal neurons[113] were grown at a cell density of 8000/cm^2^ on coverslips. Hippocampal neurons of DIV 6 were harvested on DIV 6 for imaging. On the day of harvest, the concentrations of reagents used to treat neurons were: 50mM NMDA, 30 mM KCl, 5 ng/ml leptomycin B.

## Supporting Information

S1 Fig Representative confocal images of adult MB. MBs were visualized by mCD8-GFP and stained with axonal marker Fascillin II (Fas II) antibody from 5-day old adult fly expressing indicated transgenes driven by GFP;;OK107-Gal4 under ISO or EE condition. Genotype as indicated.
(TIFF)

S2 Fig Schematic of tested regions on selected genes. (A) Schematic of selected promoter and gene coding region used for ChIP experiments. (B) Consensus sequence illustrated over selected gene targets.
(TIFF)

S3 Fig Representative immunohistochemistry staining on hippocampal neurons. Immnunostaining using Abs against Tip60 and cytoplasmic and nuclear neuronal markers reveals a cytoplasmic and nuclear distribution pattern for Tip60 in neurons, consistent to what we observe in fly neuronal circuits.
(TIFF)
