# Supplementary figures and images for: Tip60 HAT Action Mediates Environmental Enrichment Induced Cognitive Restoration
Source: PLoS One. 2016 Jul 25;11(7):e0159623. doi: 10.1371/journal.pone.0159623 (PMC4959735; doi:10.1371/journal.pone.0159623)

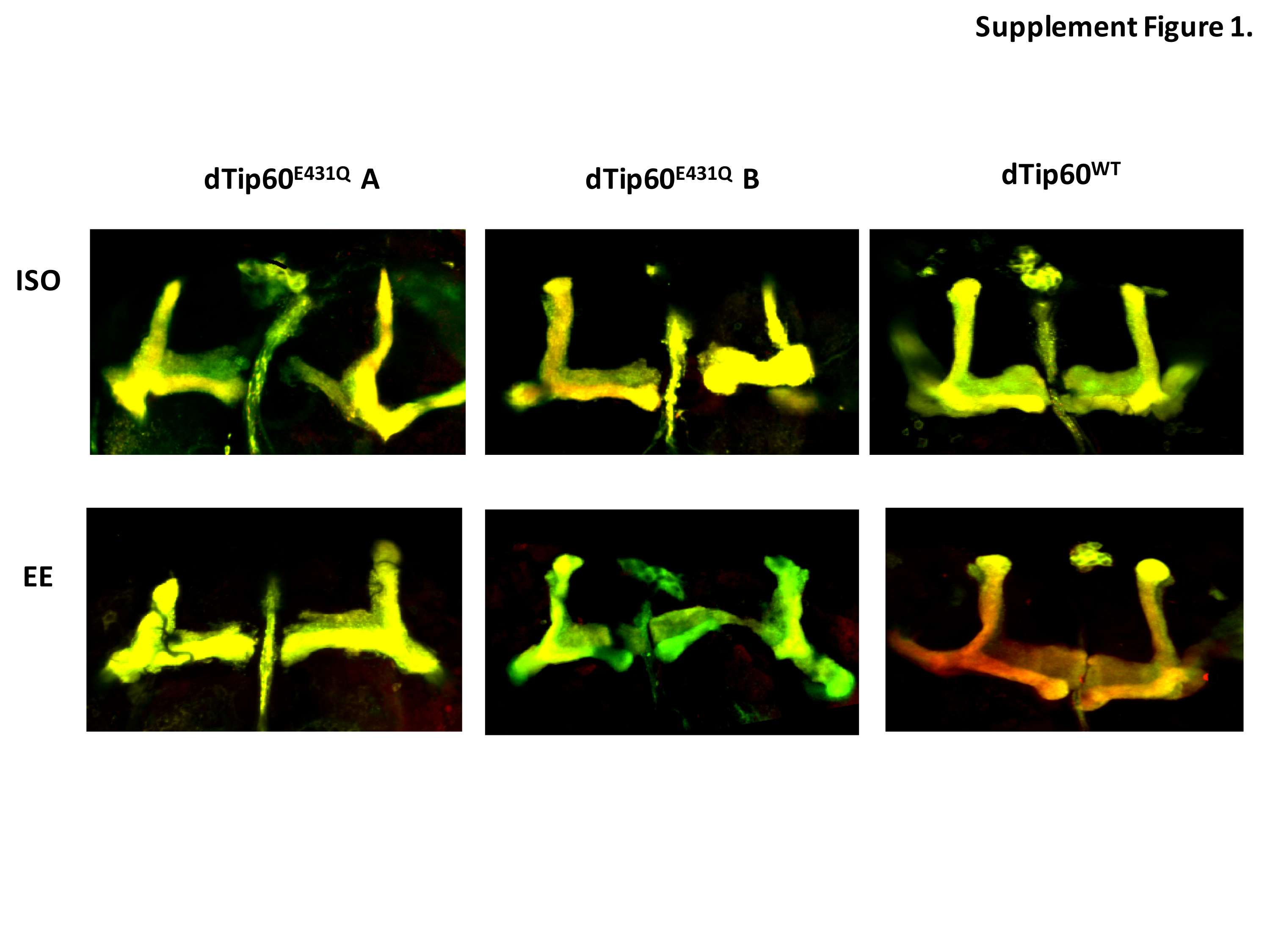

Supplement: S1 Fig — MBs were visualized by mCD8-GFP and stained with axonal marker Fascillin II (Fas II) antibody from 5-day old adult fly expressing indicated transgenes driven by GFP;;OK107-Gal4 under ISO or EE condition. Genotype as indicated. (TIFF) [file pone.0159623.s001.tiff]

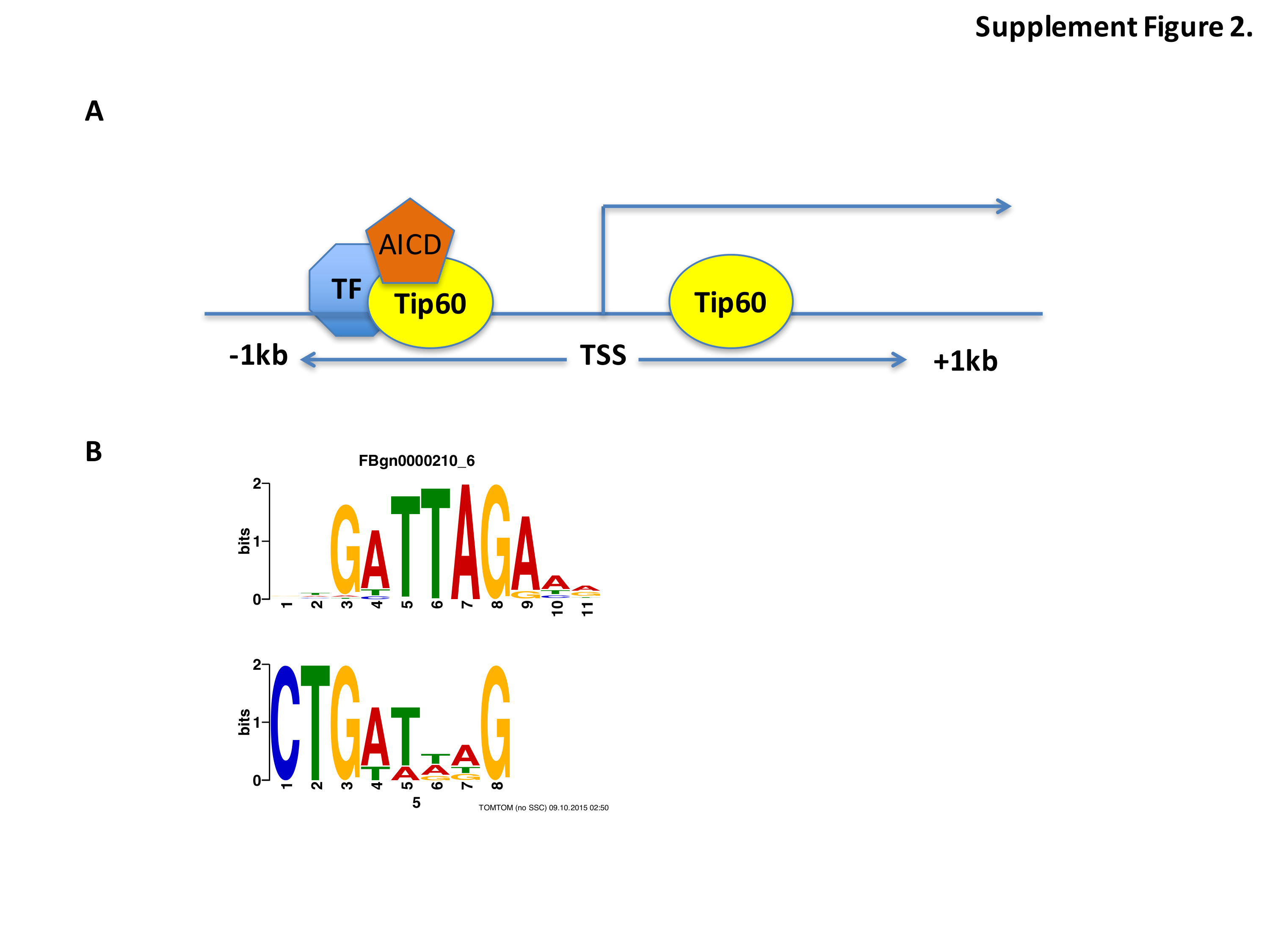

Supplement: S2 Fig — (A) Schematic of selected promoter and gene coding region used for ChIP experiments. (B) Consensus sequence illustrated over selected gene targets. (TIFF) [file pone.0159623.s002.tiff]

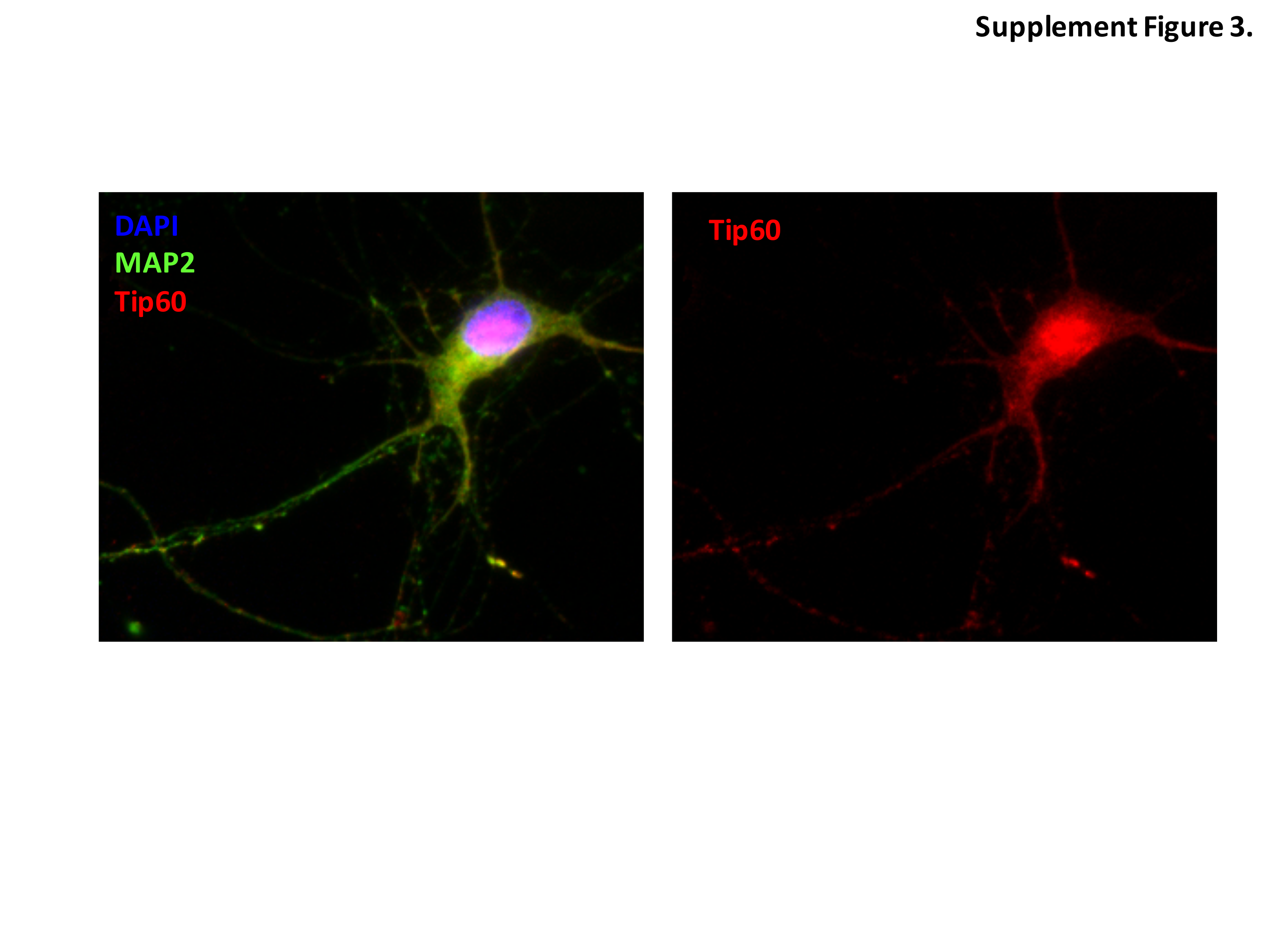

Supplement: S3 Fig — Immnunostaining using Abs against Tip60 and cytoplasmic and nuclear neuronal markers reveals a cytoplasmic and nuclear distribution pattern for Tip60 in neurons, consistent to what we observe in fly neuronal circuits. (TIFF) [file pone.0159623.s003.tiff]
